# Sensitivity of the mystacial vibrissal system of harbour seals (*Phoca vitulina*) to size differences of single vortex rings

**DOI:** 10.1242/jeb.249258

**Published:** 2025-10-01

**Authors:** Yvonne Krüger, Wolf Hanke, Lars Miersch, Guido Dehnhardt

**Affiliations:** University of Rostock, Institute for Biosciences, Sensory and Cognitive Ecology, Rostock 18059, Germany

**Keywords:** Hydrodynamic sensory system, Marine mammal, Pinnipeds, Sensory biology, Prey capture

## Abstract

Seals can detect extremely weak water disturbances with their highly sensitive vibrissae. This allows them to detect and track hydrodynamic trails that swimming prey fish inevitably leave behind. As these trails differ depending on the characteristics of the fish generating them, they may reveal information about not only the swimming direction, but also the fish species, its body shape and size, its swimming style and speed, and the time that elapsed since the fish swam by. Most fish trails provide this kind of information by consisting of a continuous three-dimensional chain of vortex rings. However, when a fish performs an escape response, it generates a very complex flow pattern consisting of three individual jets, each traveling in a different direction, with only two forming vortex rings. This particular flow pattern might camouflage the fish's actual flight direction hydrodynamically, possibly thus offering the fish a means of deceiving the predator. Identification of the three distinct structures, by comparing and analysing their size, is a feasible way to overcome the fish's deception. The aim of this study was to investigate whether harbour seals are able to disclose such a camouflage and decipher the fish's escape direction. A harbour seal was trained to differentiate two artificially created vortex rings, perceived successively by the mystacial vibrissae, based on their size. The seal was indeed able to successfully differentiate vortex rings down to a size difference of about 18 mm. In natural situations in which a fish performs an escape response, this capability should enable harbour seals to pursue the fish's actual swimming direction.

## Teaser

**Highlighted Article:** Harbour seals can analyse the size of single vortex rings in a behavioural discrimination task where a variety of pairs of differently sized vortex rings are perceived by the mystacial vibrissae.

## INTRODUCTION

Structural specialisations and an extraordinarily high degree of innervation of pinniped vibrissae (Marshall et al., 2006; Hyvärinen et al., 2009; McGovern et al., 2014; Mattson and Marshall, 2016; Dehnhardt and Hanke, 2017) form the basis for the complex sensory abilities of these aquatic mammals. Through active touch, vibrissae allow pinnipeds like walruses, harbour seals and California sea lions to identify object characteristics like size, shape and texture (Kastelein and van Gaalen, 1988; Dehnhardt, 1994; Dehnhardt and Kaminski, 1995; Dehnhardt and Dücker, 1996; Dehnhardt et al., 1998; Grant et al., 2013; Milne et al., 2021). For harbour seals and California sea lions, however, it has been shown that, in adaptation to their aquatic environment, their vibrissae also allow them to perceive extremely weak water disturbances (Dehnhardt et al., 1998; Dehnhardt and Mauck, 2008). This enables them to detect and track the hydrodynamic trails left behind by moving abiotic (Dehnhardt et al., 2001; Wieskotten et al., 2010a; Gläser et al., 2011) and biotic (Schulte-Pelkum et al., 2007) objects.

Complex, three-dimensional hydrodynamic trails are inevitably generated by fish swimming through the water column. In terms of structure, spatial arrangement and water velocity, they depend on the characteristics of the fish species, such as body shape, size, swimming style and speed (Bleckmann et al., 1991; Hanke et al., 2000; Müller et al., 2000; Hanke and Bleckmann, 2004; Standen and Lauder, 2007). For example, the width of a trail correlates with the body size of a potential prey fish (Hanke et al., 2000). Thus, a fish displays relevant information attributed to its nature just by means of the flow pattern left behind. Furthermore, the velocity gradient within the trail, as well as the overall moving direction of the generated flow pattern, reveals directional information about the swimming fish. A temporal component can also be extracted from a hydrodynamic trail because vorticity and flow velocity decay as the trail ages (Hanke et al., 2000). For some fish species, such as *Diplodus annularis* (Aleyev, 1977), *Oncorhynchus mykiss* (Blickhan et al., 1992), *Chelon labrosus* (Müller et al., 1997) and *Lepomis macrochirus* (Lauder and Drucker, 2002), it has been shown that their hydrodynamic trails consist of chains of linked vortex rings, with a central jet moving backward between the counter-rotating vortex rings. Other species, such as *Brachydanio albolineatus* (Rosen, 1959), *Acanthophthalmus kuhlii* (Rayner, 1995) and *Anguilla anguilla* (Müller et al., 2001), leave trails of single, mutually delimited vortex rings.

A very complex flow pattern results from a fish's escape response, the so-called C-start, in which the flight direction of the fish is hydrodynamically camouflaged to a substantial degree (Tytell and Lauder, 2008; Niesterok and Hanke, 2013). This camouflage is achieved by the generation of three individual jets travelling in different directions. A predator may only localise the prey fish's flight direction if it is able to analyse differences between the hydrodynamic structures, if they exist. Particle image velocimetry (PIV) of the C-start of a rainbow trout, performed by Niesterok and Hanke (2013), revealed that the individual jets generated through a C-start are indeed different from each other. The trout escaped approximately parallel to the direction of jet 1, which is generated by the tail fin and is clearly recognisable as a single vortex ring. Jet 2, generated by the body and tail fin, forms another vortex ring, and the fish tends to escape in exactly the opposite direction to this jet's travel path. Another comparably inconspicuous flow structure, jet 3, is generated by the body in the final stage of the escape response and can almost always be distinguished from these two by its irregular shape. Whereas jets 1 and 2 are clear vortex rings defined by their two counter-rotating vortices, jet 3 usually lacks these symmetrical counter-rotating vortices and consists of several sub-structures. Jets 1 and 2 are clearly distinguishable by their size difference, with jet 2 showing a larger vortex ring width. Both vortex rings contain directional information and being able to distinguish those from the irregular structure of jet 3, and to discriminate between them, should enable a predator, for example a harbour seal, to disclose the fish's camouflage and decipher its escape direction.

Psychophysical studies with harbour seals revealed that they can precisely discern various characteristics of the trail generator from its hydrodynamic trail. They determine the moving direction of a trail generator in less than 0.5 s when encountering the trail with their mystacial vibrissae (Wieskotten et al., 2010b). Furthermore, Wieskotten et al. (2011) demonstrated that seals can read the size and shape of different trail generators from their hydrodynamic trails with similar efficiency. In combination with PIV measurements of the trails used, the behavioural experiments by Wieskotten et al. (2011) suggested that the recognition of object size might be based on both the mean spatial extension of the trail as well as vortex ring size, which is in good agreement with the structure of the hydrodynamic trails of different fish species.

In a previous study (Krüger et al., 2018), we showed that the perception of a single vortex ring is sufficient for a harbour seal to derive directional information about the trail generator. Referring to the hypothesis of Wieskotten et al. (2011) regarding the correlation of vortex ring size and the size of the trail generator, as well as to the special situation of a predator when encountering the complex hydrodynamic effects of a C-start, we investigated in the present study whether a harbour seal is able to discriminate individual vortex rings based on their size. Reproducible vortex rings with distinct sizes, maximum particle velocities and travel velocities similar to those found in natural fish wakes were mechanically generated. The vortex rings were tested in pairs and the harbour seal was trained in a behavioural experiment to indicate the side on which the larger vortex ring impinged on the mystacial vibrissae.

## MATERIALS AND METHODS

### Experimental subject

The behavioural experiments were conducted with a male harbour seal (*Phoca vitulina* Linnaeus 1758), named Filou, born in 2006 in captivity. This seal was familiar with psychophysical experiments in general, and with the setup and nature of the stimuli used in this study in particular, as he was a subject in the study by Krüger et al. (2018), in which he was trained to detect single vortex rings and their travel direction by means of his mystacial vibrissae. The seal received approximately 80% of its daily diet (1.5–3 kg of freshly thawed, cut herring or sprats per day, supplemented with vitamins) as primary reinforcement during experimental sessions. At no time was the animal deprived of food. In general, one experimental session was conducted per day, 5 days a week. All experiments were conducted in accordance with the German animal protection law.

Another harbour seal (Moe), who was also a subject in the study by Krüger et al. (2018) and thus familiar with the setup and the vortex rings, was trained in this behavioural experiment. However, he only learned the experimental procedure, but not the complex task.

### Experimental setup and vortex ring generator

**Fig. 1.**
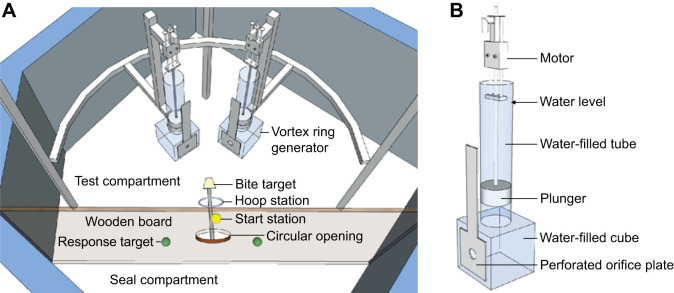
**Experimental setup (adapted from Krüger et al., 2018) to assess differentiation of vortex rings by the harbour seal (*Phoca vitulina*).** (A) Experimental setup, from a bird's eye view. The experimental pool was divided into a test compartment and a seal compartment by a wooden board. In the test compartment, two vortex ring generators (VRGs) were mounted on a semi-circular horizontal profile at a 35 deg angle on either side of the animal's underwater station. The station consisted of a fixed hoop and a bite target, which in combination ensured consistent head positioning during testing. The station was accessible to the animal through a 30 cm wide circular opening in the wooden board. Attached to the board in the seal compartment were the start station (yellow plastic sphere), where the animal had to wait prior to each trial, and the two response targets (green plastic spheres). The working platforms, where the experimenter and the assistant were positioned, consisted of two timber planks placed across the pool between the VRGs and the wooden board; these are not shown. (B) Schematic drawing of one of the two VRGs. The VRG was immersed in the pool up to the indicated water level. The motor is remote controlled (the remote and wiring of the motors to the technical equipment are not visualised) and pushes the connected plunger down towards the water-filled cube. A predefined amount of water is expelled through the aperture of the orifice plate and forms a vortex ring. The size of the circular aperture of the mounted orifice plate depicted is 4 cm, and the distance to the bite target is 30 cm.

The study was conducted at the Marine Science Center (Rostock, Germany), which is located within a marina in the Baltic Sea. The facility consists of a moored ship (60 m in length), providing office and laboratory space, and a mesh-surrounded enclosure (3500 m², 6 m maximum depth) with different floating training areas, housing the animals in natural seawater. The experimental pool (Fig. 1) was located outside on the rear deck of the ship and filled with natural sea water. It had dimensions of 2.3 m×2.3 m×1 m (L×W×D) and was surrounded by transparent Makrolon windshields on the west and north sides. The pool was accessible to the seal only during experimental sessions via a gangway connecting the animal enclosure with the ship. Not visualised in Fig. 1 are two timber planks (2.95 m long by 0.3 m wide) across the pool, which served as working platforms for the experimenter and the assistant. A vertical wooden board with a circular opening (30 cm in diameter) separated the pool into two compartments: a test compartment and a seal compartment.

Within the seal compartment, a start station (yellow plastic sphere, Fig. 1) was mounted above the circular opening of the board, where the seal could place its head just above the water surface prior to each trial. Furthermore, on either side of the circular opening, a response target (green plastic sphere, Fig. 1) was fixed to the board.

In the test compartment, an underwater station was installed right behind the circular opening of the board. This station consisted of a hoop with a diameter adjusted to the seal's neck diameter and a bite target for the seal's mouth (food-safe polyurethane). It ensured consistent and fixed head positioning during stimulus presentation. At an angle of 35 deg on either side of the animal's underwater station, custom-made vortex ring generators (VRGs) were attached to a semi-circular horizontal aluminium profile (diameter 1.8 m).

The VRGs were constructed from acrylic glass and consisted of a cube, which opened on its top side to a vertical pipe. The cube had dimensions of 20×20×20 cm, with a circular opening on the front side of 6.5 cm in diameter. The size of this opening was altered by interchangeable slip-on orifice plates with circular apertures of either 2 or 4 cm in diameter. The vertical pipe had an inner diameter of 10 cm and contained a movable plunger operated by a linear motor (LinMot, NTI AG, Spreitenbach, Switzerland). The motor implemented a predefined downward movement of the plunger, thus expelling water through the aperture of the orifice plate, which then formed into a vortex ring. Each motor was controlled individually via a remote control to create two single vortex rings successively, one from each generator. Both VRGs were oriented towards the underwater station at a distance of 30 cm between the apertures of the orifice plates and the bite target. The centre of the aperture was at the same height as the bite target, which was in the middle of the water column, 50 cm above the pool ground.

The experiment was conducted in the same setup as the previously published behavioural experiments (Krüger et al., 2018), with the only exception that both VRGs were oriented towards the animal at a fixed angle of 35 deg, so that not just one, but two vortex rings were perceived by the test animal per trial.

### Stimuli: vortex rings

Seven different vortex rings, with properties similar to those found in fish wakes, were selected for the behavioural experiments. Vortex ring properties were precisely adjustable by modifying the size of the aperture of the orifice plate (2 or 4 cm) of the VRGs and the parameters of the motor configuration (see Table 1). The latter were regulated by the software LinMot-Talk1100 (LinMot, NTI AG, Spreitenbach, Switzerland) and included the ‘acceleration’, ‘deceleration’ and ‘velocity’ of the plunger movement, and the ‘amplitude’ of the plunger displacement. The higher the ‘amplitude’, the greater the amount of water that would be expelled to form into a vortex ring. The motor parameter ‘deceleration’ (10 m s^−^²) was constant for all stimuli. The sizes, maximum flow velocities and travel velocities of the vortex rings are shown in Table 1. Five of the seven stimuli were used in the study by Krüger et al. (2018). For recognition purposes, the names of the stimuli (Table 1, leftmost column) were retained here, even though their successive order is thus no longer given. Occasionally, in some sessions, the water within the cubes was stained with uranine (Fluorescein sodium C_20_H_10_Na_2_O_5_, Kremer Pigmente GmbH & Co. KG, Germany) so that the experimenter could visualise the vortex rings. For further information on the analysis and characterisation of the vortex ring parameters via particle image velocimetry (Adrian, 1991; Willert and Gharib, 1991; Westerweel, 1997), using the software DaVis 7.2 (LaVision GmbH, Göttingen, Germany) and MATLAB 6.5 (MathWorks Inc., Natick, MA, USA), see Krüger et al. (2018).

**Table 1.** Size of the aperture of the orifice plate, motor parameters and vortex ring properties of seven vortex rings used in the psychophysical experiment

Vortex ring	Aperture (mm)	Motor parameters			Vortex ring properties						
					Size (mm)	Max. velocity (m s^−^¹)		Travel velocity (m s^−^¹)		Acceleration (m s^−^²)	Stimulus duration (s)
		Amplitude (mm)	Acceleration (m s^−^²)	Velocity (m s^−1^)		23 cm	34 cm	23 cm	34 cm		
S1	20	3.0	10	0.5	89.89±3.31	2.108	1.800	1.073	0.903	–1.574	0.108
S2	20	2.0	10	0.5	68.95±1.58	1.397	1.111	0.649	0.494	–0.858	0.180
S7	20	1.5	0.5	0.5	51.11±1.36	0.763	0.554	0.356	0.246	–0.332	0.329
S9	20	2.0	0.2	0.5	54.14±1.90	0.666	0.424	0.301	0.195	–0.270	0.389
S10	20	1.5	0.2	0.5	45.78±1.11	0.538	0.263	0.262	0.166	–0.213	0.447
S11	40	7.0	0.5	2.0	73.22±1.72	0.466	0.378	1.057	0.149	–0.052	0.635
S12	40	9.0	0.5	2.0	86.58±2.77	0.602	0.525	0.849	0.208	–0.069	0.481

The motor parameter ‘deceleration’ (10 m s^−^²) was constant for all stimuli and is not included in the table. For vortex ring size analysis, vectors with a velocity of 0.04 m s^−1^ in the cross section of the vortex ring (see fig. 3 in Krüger et al., 2018) were defined as the outer borders of the vortex ring. Vortex ring size was measured for all vector fields where the vortex ring was within the area of 200–315 mm from the aperture of the orifice plate, and the mean of these data is referred to as the mean vortex ring size. Mean values±s.d. are given. The maximum velocity was calculated by averaging the ten highest velocity vectors in each vector field within the area of 100–315 mm from the aperture and calculated by linear regression for two positions of the vortex ring (23 cm and 34 cm from the aperture), which represent the area of vibrissal stimulation. Acceleration and stimulus duration were calculated for the same area (23 cm to 34 cm). Vortex rings S1–S10 were previously analysed and used in the study by Krüger et al. (2018). Size is shown as mean±s.d.

#### Comparison with fish-generated vortex rings

The vortex rings generated by the VRGs resemble those generated by fish, as they exhibit a Rankine vortex ring form, the type that has been described in fish wakes (Blickhan et al., 1992; Müller et al., 1997; Hanke et al., 2000). Regarding vortex ring size, our set of vortex rings corresponds well with the range of sizes found in fish trails. We defined the size of our artificially generated vortex rings with respect to the sensory abilities of the harbour seal's vibrissal system. Specifically, we measured the distance between two opposite velocity values of 0.04 m s^−1^ in the cross-section of the vortex ring to define its size. This velocity value is clearly distinguishable from the background noise and falls within the perception range of the experimental animal (Dehnhardt et al., 1998). This defined width exceeds the distance between the two vortex ring cores, which is typically used in the literature to measure the size of fish-generated vortex rings. As a result, the size values found in fish literature are underestimated compared to our vortex rings. While vortex ring S2 (see Table 1) has a core-to-core diameter of 23.94 mm, its perceptible water flow exceeds this area almost threefold, reaching a vortex ring size of 68.95±1.58 mm, according to our definition.

Our largest vortex ring, S1, with a mean size of 89.89±3.31 mm (mean±s.d.) is supposedly smaller than the vortex rings shed by a 20 cm large (total body length) black surfperch (*Embiotoca jacksoni*) swimming at a speed of 1.5 body lengths per second [core-to-core radius: 32.1±0.35 mm (mean±s.e.m.)] (Drucker and Lauder, 2000). In contrast, a bluegill sunfish (*Lepomis macrochirus*) of the same size and swimming speed as the black surfperch generates vortex rings with a core-to-core radius of 15.4±0.08 mm (mean±s.e.m.), which is supposedly larger than our smallest vortex ring, S10, with a mean size of 45.78±1.11 mm (mean±s.d.). With respect to the vortex rings generated in a C-start by a rainbow trout (mean body length: 27 cm), the larger vortex ring measured 217.77±47.09 mm and the smaller vortex ring measured 143.46±30.64 mm. In this case, Niesterok and Hanke (2013) defined the size in a similar manner (with a particle velocity of 0.05 m s^−1^ in the cross-section), which allows for a valid comparison. Both vortex rings exhibit larger dimensions than our vortex rings.

### Psychophysical procedure and task

Since the animal had already performed hydrodynamic experiments in the same experimental setup (Krüger et al., 2018), it was familiar with the general experimental procedure and the nature of the stimuli. Prior to each trial, the experimenter blindfolded the seal with a visually opaque mask. The mask was made from elastic material and adapted seamlessly to the shape of the snout, covering the eyes completely. It had an opening in the front for the mystacial pads so that the mystacial vibrissae were completely unobstructed by the mask. In addition to the visual masking, auditory stimuli were masked via headphones transmitting pink noise. While the seal was pressing its snout on the start station, the assistant set up the VRGs. Depending on the stimuli required for the trial, the VRGs were supplied with orifice plates with an aperture of 2 or 4 cm (see Table 1). The orifice plates were consistently exchanged prior to each trial, even if the setting did not need to be changed in consecutive trials. The duration of the preparation of the VRGs, the acoustic noise, or water disturbance caused by removing and inserting the orifice plates could thus not be used as secondary cues by the seal. Removing the headphones signalled the seal to leave the start station, dive, stick its head through the circular opening of the wooden board and the hoop, and station itself in the test compartment by biting on the bite target. The assistant waited 10 s before generating the vortex rings by remote control, so that calm water conditions were re-established in the test compartment. The test animal had to remain stationed underwater until it had perceived two successive vortex rings, one from each VRG. As soon as the first vortex ring had impinged on the vibrissae, the second vortex ring was generated, so that the animal perceived the vortex rings in immediate succession. The animal's task was to differentiate between the vortex rings based on their size. The animal was trained to recognise the larger vortex ring, either coming from the right or the left VRG, and indicate the side on which the larger stimulus impinged on its vibrissae. Each vortex ring stimulated the ipsilateral vibrissae unilaterally. Immediately after both vortex rings had impinged on the mystacial vibrissae, the experimenter blew a short whistle, which was the seal's signal to leave the underwater station and give a response. According to a two-alternative forced choice procedure, it had to touch the right or the left response target in the seal compartment with its snout (Fig. 1). If the seal touched the corresponding response target correctly, a whistle was blown for secondary reinforcement. Thereupon, the seal surfaced, and after the mask had been removed, it was rewarded with fish.

### Stimulus combinations

In a natural C-start escape response, the flight direction of the fish is enclosed within both of the two vortex rings of jet 1 and jet 2 but not within the irregular structure of jet 3. Thus, we focused on comparing two vortex rings in the behavioural experiment. The relevant test parameter in this study was the size difference between the two vortex rings (see Table 2). To prevent the animal from responding to the velocity parameter of the perceived stimuli rather than to vortex ring size, we tested in each session a stimulus set with three combinations (see Table 3), consisting of vortex rings of different sizes and velocities. In these combinations, velocity did not consistently indicate the positive stimulus (S^+^). This way the task could only be solved by comparing the sizes of both vortex rings. The three combinations are as follows: In combination 1, the larger vortex ring (S^+^), contained higher maximum water velocities than the smaller vortex ring (S^−^). In contrast, in combination 2, S^+^ was larger but exhibited slower maximum velocities in the central jet than the negative stimulus (S^−^). The vortex rings of the third combination differed in size (S^+^>S^−^), but the maximum water velocities were almost equal when impinging on the vibrissae.

**Table 2.** Stimulus parameters

		Set 1	Set 2	Set 3
Combination 1	Mean size S^+^ Mean size S^−^ Size difference	89.89±3.31 45.78±1.11 44.11	68.95±1.58 45.78±1.11 23.17	68.95±1.58 45.78±1.11 23.17
Combination 2	Mean size S^+^ Mean size S^−^ Size difference	86.58±2.77 68.95±1.58 17.63	73.22±1.72 51.11±1.36 22.12	86.58±2.77 68.95±1.58 17.63
Combination 3	Mean size S^+^ Mean size S^−^ Size difference	73.22±1.72 45.78±1.11 27.45	86.58±2.77 54.14±1.90 32.44	73.22±1.72 45.78±1.11 27.45

The table shows the relevant parameter, namely the mean size±s.d. (in mm) of each vortex ring and the size difference (in mm) between the positive and the negative stimulus for every combination in every set. The largest size difference of 44.11 mm occurred in stimulus combination 1 in set 1, when S1 was tested against S10, and the smallest size difference of 17.63 mm was achieved by combining stimulus S12 with stimulus S2, in combination 2 in set 1 and set 3. For velocity values, refer to Table 1.

**Table 3.** Three sets of stimulus combinations used in the behavioural experiments

Combination	Set 1	Set 2	Set 3
1	S1 vs S10	S2 vs S10	S2 vs S10
2	S12 vs S2	S11 vs S7	S12 vs S2
3	S11 vs S10	S12 vs S9	S11 vs S10

Each stimulus set consists of three combinations of vortex rings. The stimulus combinations are shown in the following order: S^+^ vs S^−^. In combination 1, S^+^ is larger in size and contains higher water velocities in the central jet. S^+^ in combination 2 is larger but contains slower water velocities compared with S^−^. Combination 3 comprises a larger vortex ring for S^+^ with water velocities similar to those of S^−^.

All three combinations were tested equally often within one session per day (12 trials per combination, 36 trials in total). The side of S^+^ and whether S^+^ was generated first or second were distributed equally often and pseudorandomly within the sessions, following the principles established by Gellermann (1933), modified according to Holt and Schusterman (2002). This led to four different possible stimulus pairings per combination. Pair 1: S^+^ is perceived first from the left side, then S^−^ is perceived from the right side. Pair 2: S^−^ is perceived first from the left side, then S^+^ is perceived from the right side. Pair 3: S^+^ is perceived first from the right side, then S^−^ is perceived from the left side. Pair 4: S^−^ is perceived first from the right side, then S^+^ is perceived from the left side.

Discontinued sessions, in which the seal left the setup beyond recall before the last trial, were not finished later and were disregarded in the data analysis.

### Transfer tests

To check whether the animal had merely learned the three stimulus combinations of the first set (shown in Table 3), new stimulus combinations meeting the same criteria were tested subsequently. In set 2, a special challenge was introduced in that the formerly negative stimulus S2 (from combination 2 in set 1) contrariwise became a positive stimulus (see combination 1 in set 2). Similarly challenging, in set 3, stimulus S2 was both positive and negative depending on the combined second vortex ring.

### Control experiments

Two types of control experiments were conducted in order to exclude the possibility that the seal had used secondary cues for its decision, or a different sensory system than the mystacial vibrissae to solve the task. Both control experiments were run in the exact same manner as the test experiment.

#### Motor noise

Since the generators were triggered individually with different motor parameters, the noise produced by the motors during stimulus generation differed depending on the stimulus. In the control sessions, the same stimulus combinations were generated as in the test sessions, except that the generators were turned away from the animal. Consequently, no vortex rings were perceived by the seal's vibrissae. Nevertheless, the VRGs generated vortex rings travelling to the rear wall of the pool, thus producing the exact same noise as in the test experiment. The control sessions were conducted after data acquisition for each set of stimulus combinations and prior to testing a new set. For set 1 and set 2, each control session consisted of 24 trials without a vortex ring and 12 interspersed trials with a vortex ring perceivable by the vibrissae (36 trials per session and set, 24 control trials per set). Subsequent to the data collection for set 3, two control sessions were conducted, each consisting of 15 trials (five trials per combination) without a vortex ring and 21 trials with a vortex ring to maintain the motivation of the animal (36 trials per session, 30 control trials in total).

#### Masked vibrissae

As an additional control, the experiment was conducted in the final session with the vibrissae masked, in order to confirm that the seal mastered the task by means of its vibrissal system only. The seal's mystacial vibrissae were covered by a nylon stocking mask, which was permeable to water. The session consisted of 36 trials in total, but only half of the trials were run with masked vibrissae. For motivational purposes, 18 trials without the vibrissal cover were interspersed. Each stimulus combination was tested equally often in both masking conditions: three times with and three times without the nylon stocking mask.

## RESULTS

### Acquisition of the task: stimulus set 1

**Fig. 2.**
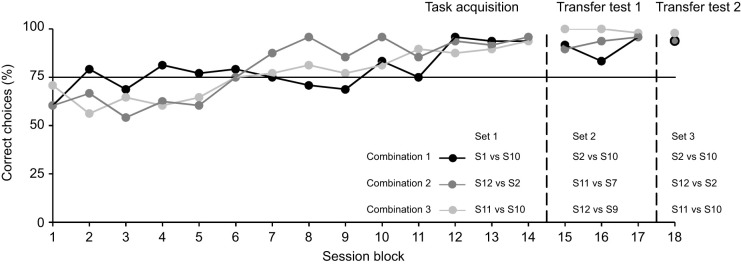
**Performance of Filou in discriminating two vortex rings differing in size.** Correct choices (in percentages) are plotted against session blocks. Each data point consists of 48 trials pooled from four sessions. The horizontal line indicates the level of significance (χ^2^-test, *P*<0.001, *N*=48). Session blocks 1 to 14 were conducted with the first set of stimulus combinations (S1 vs S10, S12 vs S2, S11 vs S10). Sessions after the first vertical line (session block 15–17) represent the first transfer test with the second set of stimulus combinations (S2 vs S10, S11 vs S7, S12 vs S9). The last session block (session block 18) consists of transfer sessions with the third set of stimulus combinations (S2 vs S10, S12 vs S2, S11 vs S10).

The seal's learning curve for distinguishing the larger vortex ring from the smaller vortex ring is shown in Fig. 2. Each session block consists of pooled data from four consecutive sessions, showing the performance for each of the three stimulus combinations (48 trials per data point). It took Filou 56 sessions (14 session blocks with 2016 trials in total) to reach the learning criterion of at least 80% correct choices for all three stimulus combinations in three consecutive session blocks, which is highly significantly above chance level (χ^2^-test, *P*<0.001, *N*=144). The largest size difference between consecutive vortex rings was 44.11 mm. The smallest size difference tested in set 1 was 17.63 mm (see Table 2). In comparison, Moe did not learn the task within 70 sessions (2520 trials) with the first set of stimulus combinations. For an additional 7 sessions (252 trials), stimulus combination set 2 was tested, without achieving an improvement in performance. Subsequently, 56 sessions (2016 trials) with two different variations, in order to achieve learning, were conducted (testing only one stimulus combination per session and even generating only the positive stimulus). A final reduction to only 20 trials per session and testing only one stimulus combination for another 21 sessions (420 trials) did not result in successful learning. In total, Moe did not learn the task within 5208 trials.

The seal's response behaviour during the learning phase was analysed. There was no response preference for a specific response target; Filou touched the right and the left response target equally often. He did not show a preference for responding to the velocity parameter of the vortex rings. However, there was a response preference in the very beginning for the vortex ring perceived second. In four out of the first eight sessions, Filou responded significantly more frequently on the corresponding response target of the vortex ring perceived last (1st session: 86.11%, 4th session: 80.56%, 5th session: 72.22%, 8th session: 80.56%). During the first three sessions of the experiment, Filou performed early starts – trials in which he left the underwater station after the perception of the first vortex ring. Those trials were discontinued and started anew (15 early starts in the first session, four early starts in the second session, one early start in the third session; no more early starts thereafter).

### Transfer test 1: stimulus set 2

Set 2 consisted of the following three new stimulus combinations, in which the former is the larger vortex ring: S2 vs S10, S11 vs S7 and S12 vs S9. When introduced to new stimulus combinations for the first time, Filou's overall performance remained at a highly significant level (never below 83.33%; χ^2^-test, *P*<0.001, *N*=48). Even though the set of stimulus combinations was unfamiliar to Filou, he responded 94.44% correctly in the very first session (χ^2^-test, *P*<0.001, *N*=36). In two of the stimulus combinations (combination 2: S11 vs S7, and combination 3: S12 vs S9), Filou responded 100% correctly (χ^2^-test, *P*<0.001, *N*=12). He only responded incorrectly twice in stimulus combination 1 (S2 vs S10) (83.33%; χ^2^-test, *P*<0.05, *N*=12). Stimulus S2 was formerly the negative stimulus in set 1 when it was combined with the larger vortex ring S12. In set 2, the vortex ring S2 became the positive stimulus in combination with S10. The largest size difference tested in set 2 was 32.44 mm, and the smallest size difference was 22.12 mm (see Table 2).

### Transfer test 2: stimulus set 3

Set 3 comprised the following three stimulus combinations: S2 vs S10, S12 vs S2 and S11 vs S10. Again, the first stimulus listed in the combination is the larger vortex ring. The three stimulus combinations in stimulus set 3 had been previously tested (for size differences, see Table 2), but not within the same set. In stimulus set 3, the vortex ring S2 was both positive and negative, depending on the combined second vortex ring. Nevertheless, Filou's performance in the very first session, when testing stimulus set 3, was highly significant with 100% correct choices for these two stimulus combinations (S2 vs S10 and S12 vs S2) (χ^2^-test, *P*<0.001, *N*=12). His performance for the third combination (S11 vs S10) was similarly high, with only one mistake, leading to 91.67% correct choices (χ^2^-test, *P*<0.01, *N*=12). The overall performance for the session block of set 3 was highly significant as well for all three combinations, with 93.75% (S2 vs S10), 93.75% (S12 vs S2) and 97.92% (S11 vs S10) correct choices (χ^2^-test, *P*<0.001, *N*=48).

### Control experiments

#### Motor noise

The data points for the control experiments, in which the generators were oriented away from the animal, are not included in Fig. 2. The control sessions were conducted after data acquisition for each set with the same stimulus combinations (for set 1, after session block 14; for set 2, after session block 17; and for set 3, after session block 18). In trials where no vortex ring impinged on the vibrissae, the performance dropped to chance level (control session set 1: 54.17%; χ^2^-test, *P=*0.68, *N*=24; control session set 2: 62.5%; χ^2^-test, *P*=0.22, *N*=24; control session set 3: 56.67%; χ^2^-test, *P*=0.465, *N*=30). The interspersed trials showed that the drop in performance was not due to motivational issues. The seal's performance remained at a highly significant level (in set 1 and 2: 100% correct choices; χ^2^-test, *P<*0.001, *N*=12; in set 3: 88.1% correct choices; χ^2^-test, *P<*0.001, *N*=42) in trials where the vortex rings stimulated the mystacial vibrissae. The very first trial of the first control session for set 1 was a control trial with the generators turned to the rear wall of the pool. After the generation of both vortex rings (not perceivable by the seal's vibrissae), Filou left the bite target and came up to the water surface without responding to one of the two targets. Filou did not show this behaviour in any of the test trials before, and it appears to be a result of a ‘missed’ stimulus, as if there were no stimuli to respond to. This trial was counted as incorrect. Furthermore, in the very first trial of the first control session for set 2, Filou again showed a behaviour most likely related to a ‘missing’ stimulus. In this case, after the short whistle blow, the signal to give a response, he did so even though no vortex rings impinged on the mystacial vibrissae. By chance, the response was correct, and the secondary reinforcer was given, signalling the proximate reward. Filou immediately left the experimental setup after the blindfold was removed, without taking the fish, a highly untypical behaviour observed only on this occasion.

#### Masked vibrissae

The control experiment with mixed trials of unmasked and masked vibrissae showed that the mystacial vibrissae are indispensable for solving the task. While the performance in the unmasked trials remained highly significant (88.89% correct choices; χ^2^-test, *P*<0.001, *N*=18), in the masked condition, the performance dropped to chance level (44.44% correct choices; χ^2^-test, *P*=0.637, *N*=18). Filou was not able to distinguish the larger vortex ring from the smaller vortex ring when a nylon stocking mask covered the vibrissae.

## DISCUSSION

### Discrimination of vortex ring size and learning speed

Compared with our previous experiments requiring the seal to derive directional information from travelling vortex rings (Krüger et al., 2018), Filou now needed considerably longer, 2016 trials in total, to learn the size discrimination task. Moe did not learn the task at all within 5208 trials. Filou and Moe both conducted the hydrodynamic experiments published in Krüger et al., 2018 and learned to indicate the side on which they had perceived a hydrodynamic stimulus within 100 trials. Both animals showed fast and discontinuous learning. The more sophisticated task, in which the animal had to detect the direction from which a single vortex ring impinged on its vibrissae and thus determine the travel direction of the vortex ring, took Filou 1260 trials to reach the learning criterion (of at least 80% correct choices for all four travel paths in three consecutive sessions). Moe was excluded from further training as he did not learn the task within the time frame Filou needed to complete data collection. Also in the present study, further training with Moe was terminated when data collection with Filou was completed. Nevertheless, if one animal is thoroughly tested, with all necessary control tests, and demonstrates the sensory and cognitive ability to solve the designed task, it is reasonable to conclude that harbour seals, in general, possess the problem-solving abilities required to perform such tasks in nature. The lack of success in Moe is not indicative of a deficit in sensory abilities, but rather reflects the complexity of the experimental task and the fact that the allocated time was not sufficient for him to learn the task. In the previous study from 2018, the response was based solely on the perception of a single vortex ring. The seal now had to adjust to the new challenge of comparing two stimuli successively. The response behaviour during the learning phase, such as the preference for the vortex ring perceived last and the early starts, can also be explained by the task learned in the former study, where only one vortex ring was presented. Furthermore, the four different pairings of each of the three stimulus combinations per stimulus set (see Materials and Methods) pose different decision criteria on their own. In addition, the animal had to learn to distinguish size as a decisive differentiating parameter from other hydrodynamic parameters that were inherent in the stimuli (the velocity within the jet of the vortex ring, the vorticity, or the travel velocity of the vortex ring; to mention just a few obvious parameters). Eventually, Filou successfully solved the task with a stable, highly significant performance. The transfer tests showed that Filou did not merely learn the different stimulus combinations, but made his decisions based on the relative size of individual vortex rings. Filou was already responding correctly in the very first trials of the new stimulus combinations. The control experiments regarding the noise of the motor demonstrated that he did not rely on any secondary cues, as he was unable to successfully complete the task when no vortex rings were available for comparison. The control experiments, where the vibrissae were masked, showed that no other sensory systems were used instead of the mystacial vibrissae in order to respond correctly. Filou was able to differentiate all vortex ring combinations tested in our study with a highly significant performance. The largest size difference tested between vortex rings was 44.11 mm, and the smallest size difference was 17.63 mm.

During each trial, the animal's head was positioned at a fixed location by a hoop station and a bite plate. This allowed for controlled unilateral stimulation of the ipsilateral mystacial vibrissae; the larger the vortex ring, the more vibrissae were stimulated. Owing to the consistent stationing of the animal, the VRGs could be accurately aligned in order to determine the travel path of the vortex ring prior to the trial. The vortex rings thus impinged on the corresponding vibrissal field and neither a head nor a vibrissal movement was essential for the animal to perceive or analyse the stimuli. The harbour seal waited motionless in its station until it was released by the short whistle blow. The animal consistently responded without hesitation, regardless of the stimulus combination.

### Does a fish escape response muddy the waters effectively?

Even though the three individual jets left behind by an escaping fish travel in different directions, Niesterok and Hanke (2013) concluded from their analyses that the flow pattern should provide an aquatic predator information about the escape direction of the fish. Statistically, jet 1 and jet 2 both contain directional information. While jet 1 travels approximately parallel to the escape direction of the fish, jet 2 travels in the opposite direction. Jet 1 and jet 2 are clearly distinguishable by their size difference. The ability to distinguish the size of two vortex rings could thus allow a predator to overcome the hydrodynamic camouflage of a prey fish's escape direction resulting from a C-start.

The mean jet width difference between jet 1 (143.46±30.64 mm) and jet 2 (217.77±47.09 mm) in the rainbow trout (mean body length: 27 cm) was about 74 mm, or 52%. Niesterok and Hanke (2013) used similar criteria to define the size of their flow structures, which makes the widths comparable. In their evaluation, particle velocity of 0.05 m s^−1^ in the cross-section of the jet was used as outer borders, as opposed to 0.04 m s^−1^ in our study. We showed that a harbour seal is able to discriminate size differences between two vortex rings as low as 18 mm, which is substantially smaller than the size difference between jet 1 and jet 2 in the flow pattern of an escaping rainbow trout with a mean body length of 27 cm. Although we did not determine a difference threshold, the seal tested in our study is at least able to differentiate a size difference of 18 mm (S2 and S12 differ by 26%), which is substantially smaller than the size difference of the two jets produced by the rainbow trout (jet 1 and jet 2 differ by 74 mm or 52%). The rainbow trout used in the analysis by Niesterok and Hanke (2013) is a good comparison model, as salmonid species of similar overall size, body and fin shape are indeed natural prey fish of harbour seals (Sharples et al., 2009).

The studies by Wieskotten et al. (2010b, 2011) have already indicated that vortex structures in general and their size in particular are an essential distinguishing feature of fish-generated hydrodynamic trails. Our previous study (Krüger et al., 2018) confirmed this by showing that seals can detect directional information from individually travelling vortex rings. The data from the present study not only show that a seal can perceive and differentiate the size of vortex rings, but, above all, that it can compare two vortex rings that impinge on the mystacial vibrissae in immediate chronological order and analyse them to make a decision regarding their differences.

In a natural predator–prey encounter, when a harbour seal pursues a continuously swimming fish, it should be able to detect and track the trail as long as it is within the perception range. If, in close proximity, the fish performs an escape response such as a C-start, it generates three distinct water movements that travel in different directions, camouflaging the actual flight direction. The harbour seal, actively swimming through the water column, can perceive the vortex rings with its vibrissae as it actively moves and adjusts its body and head through this complex flow pattern. While swimming, the unique undulating surface structure of the vibrissae enables the vibrissae to glide almost motionless through the water and optimises the signal-to-noise ratio, thus improving sensory performance (Hanke et al., 2010; Miersch et al., 2011). Our experimental design keeps the animal motionless, while two vortex rings are generated in immediate succession. The comparison of these two vortex rings regarding their size, even without the natural dynamic movements of the seal, offers a controlled yet meaningful way to examine the predator's ability to quickly interpret the sensory data it receives. A flow pattern from a C-start escape is a distinct natural situation in which a quick comparison of vortex sizes is necessary to discern the correct direction to pursue. Our data strongly indicate that a seal would not be misled by the different jets generated during a fish's escape response. The seal in our experiment demonstrated its ability to analyse the size difference between two vortex rings and should thus be able to recognise the larger vortex ring inherent in a C-start flow pattern. This enables the harbour seal to discern the crucial vortex ring and ultimately track the escape direction of the fish. Although the experimental design does not fully replicate the complex natural environment, it offers valuable insights into the seal's quick sensory and cognitive processing and decision making, and serves as a useful model for understanding the core mechanisms involved in pursuit decisions. Further investigations into the harbour seal's sensory ability and behaviour while actively swimming and under different natural flow conditions would be of great interest.
